# Synergizing breeding strategies via combining speed breeding, phenotypic selection, and marker-assisted backcrossing for the introgression of *Glu-B1i* in wheat

**DOI:** 10.3389/fpls.2024.1402709

**Published:** 2024-05-28

**Authors:** Jin-Kyung Cha, Hyeonjin Park, Youngho Kwon, So-Myeong Lee, Seong-Gyu Jang, Soon-Wook Kwon, Jong-Hee Lee

**Affiliations:** ^1^ Department of Southern Area Crop Science, National Institute of Crop Science, Rural Development Administration, Miryang, Republic of Korea; ^2^ Department of Plant Bioscience, Pusan National University, Miryang, Republic of Korea

**Keywords:** wheat, speed breeding, speed vernalization, marker-assisted backcrossing, molecular breeding, *Glu-B1i*

## Abstract

Wheat is a major food crop that plays a crucial role in the human diet. Various breeding technologies have been developed and refined to meet the increasing global wheat demand. Several studies have suggested breeding strategies that combine generation acceleration systems and molecular breeding methods to maximize breeding efficiency. However, real-world examples demonstrating the effective utilization of these strategies in breeding programs are lacking. In this study, we designed and demonstrated a synergized breeding strategy (SBS) that combines rapid and efficient breeding techniques, including speed breeding, speed vernalization, phenotypic selection, backcrossing, and marker-assisted selection. These breeding techniques were tailored to the specific characteristics of the breeding materials and objectives. Using the SBS approach, from artificial crossing to the initial observed yield trial under field conditions only took 3.5 years, resulting in a 53% reduction in the time required to develop a BC_2_ near-isogenic line (NIL) and achieving a higher recurrent genome recovery of 91.5% compared to traditional field conditions. We developed a new wheat NIL derived from cv. Jokyoung, a leading cultivar in Korea. Milyang56 exhibited improved protein content, sodium dodecyl sulfate-sedimentation value, and loaf volume compared to Jokyoung, which were attributed to introgression of the *Glu-B1i* allele from the donor parent, cv. Garnet. SBS represents a flexible breeding model that can be applied by breeders for developing breeding materials and mapping populations, as well as analyzing the environmental effects of specific genes or loci and for trait stacking.

## 1 Introduction

Wheat (*Triticum aestivum* L.) serves as the primary ingredient in diverse products, including breads, noodles, and cookies. It also contributes to the human diet as the third most important cereal crop in terms of total production (Shewry and Hey, 2015; Kiszonas and Morris, 2018; Igrejas and Branlard, 2020). Numerous breeding efforts have been conducted to enhance productivity, pest and disease resistance, and end-use quality. However, breeders face persistent pressure to expedite the development of new cultivars in response to challenges such as climate change and escalating global demand owing to population growth (Hickey et al., 2019). Consequently, various breeding strategies, including rapid generational advancement, phenotypic selection (PS), and molecular breeding, have been developed and deployed to enhance breeding efficiency (Pandey et al., 2022).

The speed breeding (SB) system, which utilizes an extended 22-h photoperiod, has enabled the rapid advancement of wheat generation, allowing for the production of up to six generations of spring wheat (Ghosh et al., 2018; Watson et al., 2018). The speed vernalization (SV) system, with a higher vernalization temperature of 8–10°C and a 22-h photoperiod, has facilitated up to five generations for both spring and winter wheat (Cha et al., 2022b). Subsequently, a modified speed breeding system (mSB), combined with SV, was confirmed to be suitable for practical use in large breeding populations (Cha et al., 2023b). The mSB system reduces the energy input by 80% and maximizes space utilization by harnessing natural sunlight in conventional glasses instead of relying solely on artificial lights. PS for agronomic traits and multiple disease resistance can also be effectively conducted while accelerating generations under SB or mSB conditions (Hickey et al., 2012; Dinglasan et al., 2016; Riaz et al., 2016; Cha et al., 2023b). These series of SB systems are believed to accelerate the development of breeding materials, as well as the overall wheat research by facilitating the creation of mapping populations.

Although SB systems contribute to reduced breeding times, molecular breeding — including marker-assisted selection (MAS) and marker-assisted backcrossing (MABC) — has emerged as a powerful tool for the introgression of targeted genes or major effect Quantitative trait loci (QTLs) into elite varieties (Varshney et al., 2020). Widely adopted in global wheat breeding programs (Gupta et al., 2010), molecular breeding minimizes the population size and breeding efforts by enabling the selection of lines carrying target genes or loci before harvesting (Varshney et al., 2021). Several successful applications of MAS and MABC have been observed, particularly in introgressing disease resistance, such as powdery mildew, stripe, and stem rust, and improving quality traits, such as high-molecular-weight glutenin subunits (HMW-GS) and grain protein content (Randhawa et al., 2019; Jia et al., 2020; Zheng et al., 2020; Gupta et al., 2022). Among the many types of molecular markers, the kompetitive allele-specific polymerase chain reaction (KASP) genotyping technology has gained prominence in crop breeding programs because of its high throughput, time-saving attributes, and cost-effectiveness (Rasheed et al., 2016; Bohar et al., 2020; Kaur et al., 2020; Maccaferri et al., 2022). KASP assays are useful in routine breeding applications and aid in background screening during MABC, thereby reducing the need for excessive backcrossing by selecting candidate lines with the highest recurrent genome recovery (Kang et al., 2019).

When rapid generation advancement systems are combined with molecular breeding, both breeding speed and efficiency evidently increase (Ahmar et al., 2020; Cha et al., 2023a). While numerous theoretical models have proposed combinations of SB and molecular breeding methods (Hickey et al., 2019; Voss-Fels et al., 2019; Varshney et al., 2021; Samantara et al., 2022), real-world cases demonstrating the advantages and disadvantages as well as the efficiency and improvement points of applying this theory in practical wheat breeding systems are lacking. In this study, we demonstrated a synergized breeding method (SBS) that integrates SB, PS, and MABC to introgress the *Glu-B1i* allele into one of Korea’s leading bread wheat cultivars, Jokyoung. The *Glu-B1i* allele, which belongs to the HMW-GS group, has beneficial effects on bread-making quality, including higher protein content and gluten strength, good extensibility, and increased bread loaf volume (Guo et al., 2019; Guzmán et al., 2022; Cha et al., 2022a). This study comprised the entire process, from artificial crossing to field trial evaluation. We aimed to illustrate how SB systems can effectively synergize with modern breeding technologies, thereby making them more accessible to breeders worldwide.

## 2 Materials and methods

### 2.1 Plant materials

Two wheat cultivars, sourced from the Gene Bank of the National Institute of Agricultural Sciences, Rural Development Administration (RDA), Republic of Korea (ROK), were employed to implement the synergized breeding strategy (SBS). The Korean hard white spring wheat cv. Jokyoung (IT 213249) served as the recurrent parent (Kang et al., 2006), whereas Canadian hard red spring wheat cv. Garnet (IT 205495) was used as the donor parent (McCallum and DePauw, 2008). The target gene of Garnet was *Glu-B1i*, located on chromosome 1B, which enhances bread quality by encoding the high-molecular-weight glutenin subunit (HMW-GS) 17 + 18 in wheat (Payne et al., 1987; Jang et al., 2017; Guo et al., 2019; Shin et al., 2020; Guzmán et al., 2022). Jokyoung is a leading bread wheat cultivar in Korea that harbors *Glu-B1b*, which encodes the HMW-GS 7 + 8 (Jang et al., 2017).

### 2.2 The synergized breeding strategy

**Figure 1:**
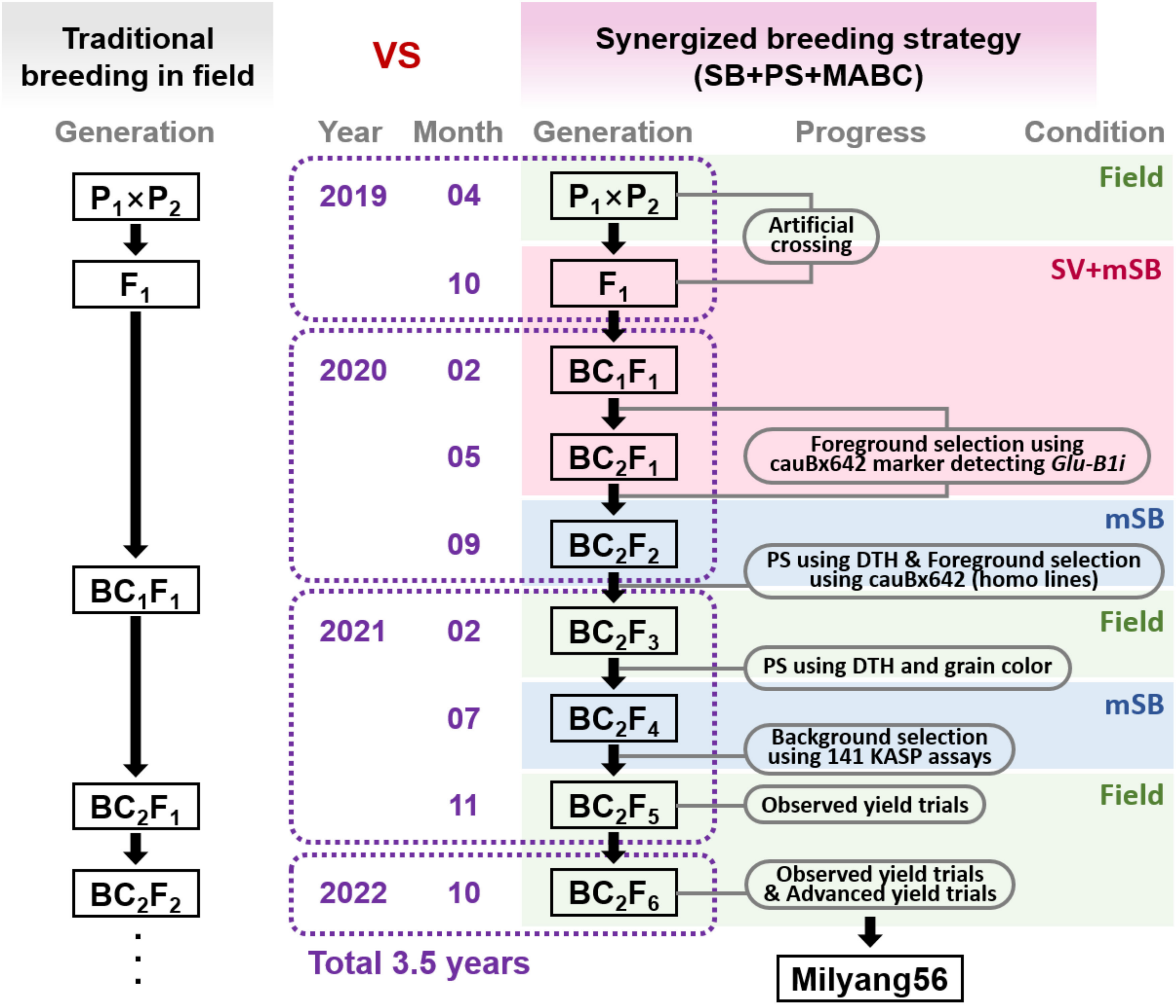
Synergized breeding strategy compared to traditional breeding method. The diagram represents the development of Milyang56 (Jokyoung*3/Garnet) by combining the speed breeding system (SB), phenotypic selection (PS), and marker-assisted backcrossing (MABC). mSB: modified speed breeding condition (22 h light, 2 h dark, 25 ± 3 °C in glasshouse); SV+mSB: speed vernalization (22 h light, 2 h dark in 8 °C for 4 weeks), then moved to the mSB condition; DTH: days to heading.

The synergized breeding strategy (SBS) incorporated three breeding tools: speed breeding (SB) system, phenotypic selection (PS), and marker-assisted backcrossing (MABC). The SBS scheme is illustrated in **Figure 1**. An initial artificial cross between Jokyoung and Garnet was conducted under field conditions. Two rounds of backcrossing with Jokyoung were performed under modified speed breeding combined with speed vernalization (SV+mSB), as described by Cha et al (Cha et al., 2023b). The BC_2_F_2_ population was evaluated under mSB conditions, which enabled visual phenotypic selection based on the difference in days to heading (DTH) between the two parental lines. BC_2_F_3_ lines were planted in the field during the spring season, and a second visual phenotypic selection was conducted considering DTH and grain color. Molecular markers were applied to the BC_1_F_1_, BC_2_F_1_, BC_2_F_2_, and BC_2_F_4_ generations to select lines closely resembling Jokyoug genetically and harboring the *Glu-B1i* gene from the donor parent. The foreground selection marker, cauBx642, was used in BC_1_F_1_, BC_2_F_1_, and BC_2_F_2_ to identify lines carrying *Glu-B1i* as heterozygous (BC_1_F_1_ and BC_2_F_1_) or homozygous (BC_2_F_2_). Background selection at BC_2_F_4_ was aimed at assessing the recurrent genomic background recovery. All BCnF_1_ plants were cultivated in 12-cm pots, which provide a larger number of spikes and grains (Cha et al., 2020), while plants from the BC_2_F_3_ generations were sown in 72-cell trays.

### 2.3 Genotyping with molecular markers

Genomic DNA was extracted from fresh seedling leaves of each plant using the MagMax DNA Multi-Sample Kit (Applied Biosystems, Woburn, MA, USA) and Kingfisher DNA extraction machine (Thermo Fisher Scientific, Waltham, MA, USA) following the manufacturer’s instructions. For foreground selection, the cauBx642 marker was used, as reported by Xu et al (Xu et al., 2008). The polymerase chain reaction (PCR) was carried out under the following conditions: initial denaturation at 95°C for 5 min, followed by 35 cycles of denaturation at 94°C for 30 s, annealing at 59°C for 30 s, and extension at 72°C for 30; and final extension at 72°C for 5 min. PCR products were separated by electrophoresis on a 3% agarose gel at 200 V for 45 min.

For background selection, a total of 731 kompetitive allele-specific polymerase chain reaction (KASP) assays were used to identify the genetic background of the developed backcrossed lines. Among these 731 assays, 155 assays referred to CIMMIYT wheat molecular genetics (Dreisigacker et al., 2016), 70 assays to Rasheed et al (Rasheed et al., 2016), and the rest to the CerealsDB core marker set (Wilkinson et al., 2012). The PCR conditions were as follows: denaturation at 95°C for 15 min; 10 touchdown cycles at 95°C for 20 s, annealing starting at 65°C and decreasing by 1°C per cycle for 25 s; additional 30 annealing cycles at 95°C for 10 s and 57°C for 1 min; and final extension at 72°C for 5 min. Fluorescence detection and data analysis were performed using a QuantStudio3 real-time PCR instrument and QuantStudio Design & Analysis Software v1.5.1 (Applied Biosystems, Carlsbad, CA, USA).

### 2.4 Phenotyping and field tests

All glasshouse-based and field tests were conducted at the National Institute of Crop Science (NICS), RDA, Miryang, ROK (35°29’32.9” N, 128°44’33.4” E). Agronomic traits, including days to heading (DTH), days to maturity (DTM), culm length (CL), spike length (SPL), number of tillers per plant (TN), number of grains per spike (GN), thousand-grain weight (TGW), test weight (TW), and grain yield (GY), were assessed following the RDA Standard Evaluation Manual for Agricultural Experiments and Research (RDA, 2012).

In the field, the BC_2_F_3_ lines were planted in late February 2021, whereas the BC_2_F_5_ lines were sown in early November 2021, representing the spring and winter growing seasons, respectively. BC_2_F_5_ lines underwent observed yield trials (OYT) in 2021, and BC_2_F_6_ lines were evaluated in both OYT and advanced yield trials (AYT) in 2022. For the OYT tests, lines were hill-seeded in the field with a spacing of 30 × 12 cm between and within the lines (a seed per hill). For the AYT tests, a rate of 14 kg/10a for each line was sown in the field as drill seeding, with 5 m rows and 30 cm spacing between rows. Fertilizer consisting of 9.1 kg N, 7.4 kg P_2_O_5,_ and 3.9 kg K_2_O per 10 a was applied in all field tests.

### 2.5 Flour quality evaluations

To analyze flour quality, the OYT and AYT lines were milled using a Brabender Quadurmat Junior mill (CW Brabender Instruments, Inc., South Hackensack, NJ, USA) and a Buhler MLU 202 laboratory mill (Bühler AG, Uzwil, Switzerland), respectively.

HMW-Gs were extracted from the flour and identified using a lab-on-a-chip (Agilent 2100 bioanalyzer & protein 230 kit, Agilent Technologies, Waldbronn, Germany), following the series of processes proposed by Shin et al (Shin et al., 2020).

Flour protein content was determined using LECO FP628 (Laboratory Equipment Co., St. Joseph, Mich., USA) with a nitrogen factor of 5.7. The gluten content and index were measured using a Glutomatic 2200 (Perten Instruments AB, Sweden). The SDS sedimentation value was determined following the method proposed by Axford et al. (1979), with a modified sample amount of 3 g. All experiments, including bread-making quality, were conducted in accordance with the American Association of Cereal Chemists (AACC) methods (AACC, 2010), and the experimental results were standardized based on 14% moisture content.

### 2.6 Statistical analysis

RStudio (version 1.4.1717; RStudio, PBC, Boston, MA, USA) was used for all statistical analyses. Chi-square tests, analysis of variance, Duncan’s multiple range tests, and t-tests were performed using the ggplot2 and agricolae packages.

## 3 Results

### 3.1 The SBS reduced the time required to develop NILs by 53%

SBS was executed according to the scheme shown in **Figure 1**. The F_1_ seeds were obtained from a cross between cv. Jokyoung and cv. Garnet under field conditions were planted under SV+mSB conditions in October 2019. Jokyoung was subjected to two rounds of backcrossing under SV+mSB conditions, followed by two rounds of foreground selection using the cauBx642 marker to detect the *Glu-B1i* allele.

**Figure 2:**
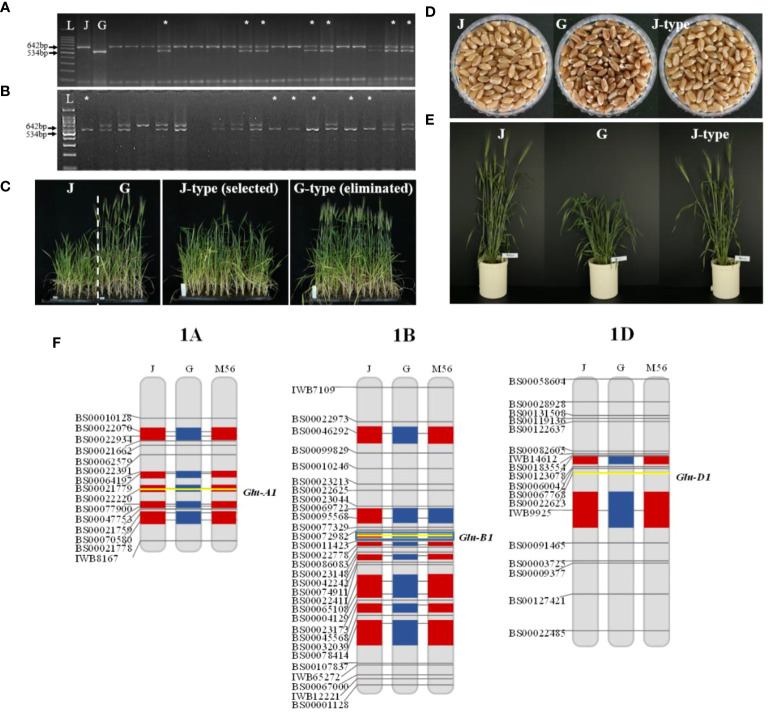
Genotypic and phenotypic selection in each generation. Foreground selection of *Glu-B1i* with cauBx642 marker in **(A)** BC_2_F_1_ and **(B)** BC_2_F_2_. Phenotypic selection in **(C)** BC_2_F_2_ families and **(D, E)** BC_2_F_3_ individuals. Background selection in **(F)** BC_2_F_4_. L: 100bp ladder marker, J: Jokyung, G: Garnet, *: Selected individuals.

The first backcross commenced in December 2019, and BC_1_F_1_ seeds were planted in February 2020. Of the six BC_1_F_1_ plants, three that exhibited the heterozygous *Glu-B1i* allele were selected for the second backcrossing (**Figure 1**, **Table 1**). A total of 31 BC_2_F_1_ seeds were sown in May 2020. Among these, 13 plants carried the *Glu-B1i* allele heterozygously and were harvested by the family for the next generation (**Figure 2A**, **Table 1**).

**Table 1:** Chi-square tests of the backcrossed populations for introducing the *Glu-B1i* allele into cv. Jokyoung.

Crossing no.	Generation	Total number of tested plants	Observed genotype frequencies			X2-test		
			Jokyoung	hetero	Garnet	Expected value	X2	P-value
YW3214	BC_1_F_1_	6	3	3	0	1:1	0.00	1.00
YW3215	BC_2_F_1_	31	18	13	0	1:1	0.81	0.37
YW3215	BC_2_F_2_	692	173	324	195	1:2:1	4.20	0.12

BC_2_F_2_ plants were cultivated in September 2020 under mSB conditions for phenotypic selection (PS) based on days to heading (DTH). As DTH varied between Jokyoung and Garnet under mSB conditions, PS was conducted within the BC_2_F_2_ populations (**Figure 2C**, **Supplementary Table S1**). Of the 31 BC_2_F_2_ families derived from each BC_2_F_1_ individual plant, 12 BC_2_F_2_ families with comparable DTH to Jokyoung and confirmed to carry *Glu-B1i* through bulked segregant analysis were chosen (**Supplementary Table S1**). Individuals from each family were assessed using the cauBx642 marker. Among the 692 individuals evaluated, 195 lines harboring homozygous *Glu-B1i* were selected for subsequent generations (**Figure 2B**, **Table 1** and **Supplementary Table S1**).

A total of 133 BC_2_F_3_ lines, yielding a sufficient quantity of seeds for field trials, were planted in the field in February 2021. PS was conducted based on the differences in DTH and grain color between the two parental lines. Consequently, 27 lines showing a heading date similar to Jokyoung (May 6) and white grains were selected (**Figures 2D, E**, **Supplementary Figure S1**). Subsequently, the 27 selected BC_3_F_4_ lines were grown under mSB conditions for background selection.

Of the 731 KASP assays collected, 141 exhibited polymorphisms between the two parents (**Supplementary Table S2**). Initially, 27 BC_3_F_4_ lines were assessed using 101 KASP assays. Sixteen lines, showing more than 85% recurrent genome recovery and more than 95% homozygosity, were identified. Among these, eight lines with the highest recurrent genome recovery and homozygosity underwent further testing with an additional 40 KASP assays (**Table 2**, **Supplementary Figure S2**). As a result of testing a total of 141 polymorphic KASP assays, these eight lines showed an average recurrent parent genome recovery of 83.9% (79.4–91.5%). These eight lines were evaluated as observed yield trial (OYT) in November 2021, and NIL-1 demonstrated the highest background genome recovery (91.5%) and similar agronomic traits, including heading date, maturity date, culm length, and other agronomic characteristics, compared to Jokyoung while carrying the introduced *Glu-B1i* allele from Garnet (**Figure 2F**, **Supplementary Table S3**, **Supplementary Figure S3**, and **Supplementary Figure S4**). Implementing SBS, from the initial crossing to the planting of the BC_2_F_6_ generation as OYT, took only 3.5 years, whereas it would have taken 7.5 years with conventional breeding methods, consequently reducing the time by 53%.

**Table 2:** Average recurrent genomic background recovery of eight Jokyoung-NIL-candidate lines in observed yield trials.

Chromosome	No. of assays showed the same genotype with cv. Jokyoung								
	Total	NIL-1	NIL-2	NIL-3	NIL-4	NIL-5	NIL-6	NIL-7	NIL-8
1A	9	9	9	9	6	9	9	5	5
1B	11	7	6	7	6	6	6	6	6
1D	2	2	2	0	2	0	0	1	2
2A	10	9	10	9	9	9	10	10	9
2B	3	3	3	3	3	3	3	3	3
2D	2	2	2	1	1	1	2	2	1
3A	11	11	10	11	11	11	11	11	11
3B	12	12	10	11	12	11	11	11	11
3D	1	1	1	1	1	1	1	1	1
4A	10	10	6	10	10	10	10	5	6
4B	9	9	8	9	7	9	9	7	8
4D	4	4	4	4	4	4	4	4	4
5A	12	12	11	12	12	12	12	12	12
5B	8	7	7	4	6	8	4	6	6
5D	4	3	3	4	3	3	3	2	2
6A	11	11	10	11	11	10	11	10	10
6B	7	6	0	3	5	5	4	6	7
6D	3	1	1	1	1	1	1	1	1
7A	3	3	3	2	3	2	3	3	3
7B	5	5	5	5	4	5	4	5	5
7D	4	2	1	2	1	2	2	1	1
Average recurrent parent genome recovery (%)	100	91.5	79.4	84.4	83.7	86.5	85.1	79.4	80.9
Hetero or missing (%)		0.0	7.1	1.4	4.3	2.1	4.3	9.9	7.1

### 3.2 Introduction of *Glu-B1i* increased bread-making quality without change in major agronomic traits

**Figure 3:**
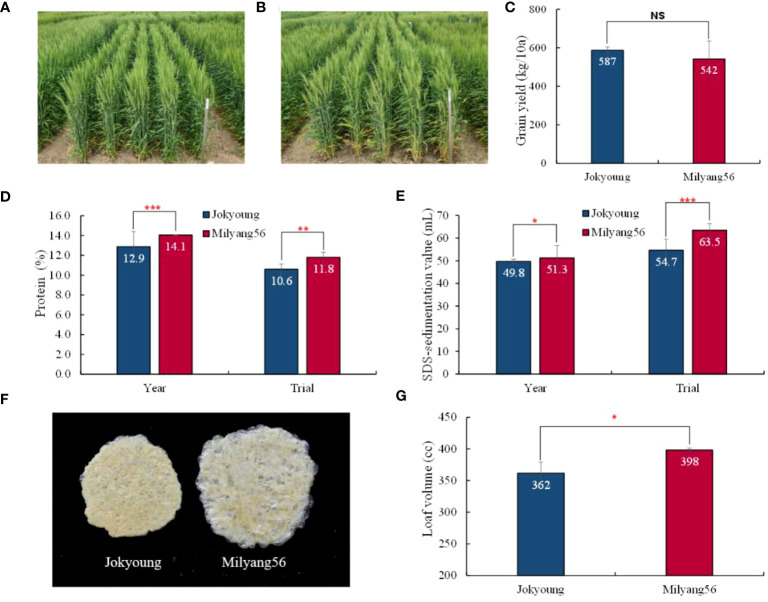
Comparison of plant phenotype and flour quality between Jokyoung and Milyang56 (Jokyoung-*Glu-B1i*-NIL) in field conditions. Phenotype of **(A)** Jokyoung and **(B)** Milyang56; Comparison of **(C)** grain yield in advanced yield test (AYT). For the comparison of **(D)** protein content, **(E)** SDS-sedimentation value, ‘Year’ represents the average performance in observed yield trials (OYT) over two years (2022–2023), while the ‘Trial’ signifies the average scores in the OYT and AYT in 2023. **(F)** dry gluten extracted from each 10 g-flour and **(G)** loaf volume tested in AYT. NS: no significant difference, * *P*<0.05, ** *P*<0.01, and *** *P*<0.001 significant difference according to t-test.

NIL-1 was designated Milyang56 and compared with the recurrent parent Jokyoung across two tested years (2021 and 2022) and trial methods (OYT and advanced yield trial(AYT)). A two-year OYT test was conducted to assess the annual variation. Overall, no significant differences were observed between Jokyoung and Milyang56 in most major agronomic traits, including DTH, days to maturity (DTM), culm length (CL), number of grains per spike (GN), and thousand-grain weight (TGW), across tested years and trial methods (**Figures 3A, B**, **Table 3**, **Supplementary Tables S4–S6**). While most agronomic traits exhibited significant differences due to environmental variations, such as the test year and trial method, there were no significant differences between Jokyoung and Milyang56. Although Milyang56 showed a shorter spike length than Jokyoung across both tested years and trial methods, no significant differences were observed in TGW and grain yield between Jokyoung and Milyang56 (**Figure 3C**, **Table 3** and **Supplementary Table S5**).

**Table 3:** Comparison of Jokyoung and Milyang56 with the average performance in observed yield trial and advanced yield trial (AYT) in 2023.

Name	DTH	DTM	CL (cm)	SPL (cm)		TN (no./10a)		GN (no./spike)		TGW (g)	TW (g/L)		GY (kg/10a)		PC (%)		DGC (%)	GI		SDSS (mL)	
Jokyoung	169	47	77	9.1		787		34.2		49.8	810		587		10.6		9.2	80.5		53.3	
Milyang56	168	47	76	8.3	***	788	ns	31.6	*	49.2	820	***	542	ns	11.8	**	9.9	94.9	**	61.6	***

DTH, days to heading; DTM, days to maturity; CL, culm length; SPL, spike length; TN, tiller number; GN, grain number; TGW, thousand-grain weight; TW, test weight; GY, grain yield; PC, protein content; DGC, dry gluten content; GI, gluten index; SDSS, SDS-sedimentation value. For TN and GY, t-tests were performed using the data from AYT. ns: no significant difference, * *P*<0.05, ** *P*<0.01, and *** *P*<0.001 significant difference according to t-test.

When the grains were milled, and the flour was tested, the protein content and SDS sedimentation value of Milyang56 were significantly higher than those of Jokyoung, irrespective of the tested years or trial methods (**Figures 3D, E**). Additionally, the gluten index tended to be higher in Milyang56, although no statistically significant difference was observed in the annual test (**Figure 3F**, **Supplementary Table S5** and **Supplementary Table S6**). As a result of introgressing the *Glu-B1i* allele without altering the agronomic traits, the bread loaf volume of Milyang56 increased by 9.9% compared to that of Jokyoung (**Figure 3G**).

## 4 Discussion

### 4.1 Flexible utilization of a series of SB combined with other breeding techniques in conventional wheat breeding programs

Our study demonstrated how a series of SB methods could be combined with other breeding techniques to form an upgraded and realized model within breeding programs. It showed breeders how to choose and apply proper generation acceleration systems according to the breeding purpose and characteristics of the breeding materials. Both the mSB and SV+mSB systems were utilized to develop a new wheat line, “Milyang56”. The mSB system notably reduced the DTH of spring wheat cultivars; however, differences in DTH between different cultivars still existed (Ghosh et al., 2018; Watson et al., 2018). Therefore, phenotypic selection was conducted using this difference point in the BC_2_F_2_ populations. In contrast, the SV+mSB condition was used in two rounds of backcrossing to exploit its ability to ensure a uniform DTH, enabling artificial crossing between parents with diverse genetic backgrounds (Cha et al., 2022b). The series of SB systems was also naturally combined with field tests, along with breeding purposes and schedules.

Molecular breeding methods, such as MAS and MABC, were also applied under mSB and SV+mSB conditions to enhance both the speed and efficiency of breeding. We demonstrated a real-world case of the SB + PS + molecular breeding model named SBS. Compared to traditional breeding methods under field conditions, SBS reduced breeding time by 53% for developing the BC_2_F_6_ NIL. Foreground selection was conducted for BC_1_F_1_ and BC_2_F_1_, and background selection was conducted for the BC_2_F_4_ generation. Milyang56 showed 91.5% recurrent parent genome recovery with *Glu-B1i* introgression from the donor parent despite only two rounds of backcrossing. The theoretical average percentages of recurrent parent genome recovery in the BC_2_ and BC_3_ generations were 87.5 and 93.8%, respectively. However, these percentages are only achieved with large populations and are usually lower in the smaller population sizes typically used in actual breeding programs (Collard et al., 2005). For example, the average genome recovery was reported to be 92.2% despite both foreground and background selection being conducted in the BC_1_F_1_ and BC_2_F_1_ generations of maize (Babu et al., 2005; Hasan et al., 2015). Although only one background selection was conducted in our study, a higher genome recovery than the theoretical average was retained because of the complementary PS.

However, opportunities exist to conduct MABC more efficiently. The accuracy of individual selection was improved when background selection was conducted in the lower generations, BC_2_F_1_ and BC_2_F_2_, rather than BC_2_F_4_. A NIL with a 96.2% recurrent genome recovery was developed as a result of background selection, with 71 assays for BC_2_F_1_ and six assays for the BC_2_F_2_ generation in rice (Kang et al., 2019). In practice, most MABC studies have conducted background selection for BC_1_F_1_, BC_2_F_1,_ and BC_2_F_2_ (Vishwakarma et al., 2014; Rai et al., 2018; Bellundagi et al., 2022).

Background selection in early generations combined with PS may significantly increase recurrent genome recovery with fewer backcrossings compared to traditional methods. To conduct genotyping in a limited time (1–2 months before harvesting) in a series of SB conditions, breeders must be able to analyze the genotype of a large amount of breeding material simply and quickly. Therefore, we utilized KASP assays, which are highly accurate, inexpensive, and flexible (Yang et al., 2019). Different types of molecular markers can be utilized for both foreground and background selection, according to the breeder’s conditions.

### 4.2 SBS facilitates comparing the effects of specific genes or loci in various environments

HMW-GS *Glu-B1i* is known to enhance bread quality by increasing protein content, gluten strength, and gluten extensibility (Guo et al., 2019; Guzmán et al., 2022). However, environmental conditions affect the allelic performance of HMW-GSs in terms of the end-use quality of wheat (Kroupina et al., 2023). In Korea, *Glu-B1i* has been reported to have higher protein content and SDS sedimentation values than other alleles in 180 wheat varieties carrying various Glu-1 alleles (Cha et al., 2022a). However, important agronomic traits, especially DTH and DTM, were diverse in the varieties analyzed in this study, and no previous study has investigated the effects of *Glu-B1i* on similar genetic backgrounds in Korea. Therefore, we developed Jokyoung-NIL, Milyang56 and compared the effect of the *Glu-B1i* allele on end-use quality. Our study confirmed the positive effect of *Glu-B1i* on bread-making quality, along with previous studies using the SBS method and an extremely shortened breeding period. This method would facilitate the comparison of the effects of specific genes or loci in various environments by quickly developing NILs, and it can be utilized in gene stacking.

## Data availability statement

The raw data supporting the conclusions of this article will be made available by the authors, without undue reservation.

## Author contributions

J-KC: Conceptualization, Data curation, Investigation, Writing – original draft, Writing – review & editing. HP: Formal analysis, Investigation, Writing – review & editing. YK: Formal analysis, Investigation, Writing – review & editing. S-ML: Formal analysis, Investigation, Writing – review & editing. S-GJ: Data curation, Investigation, Writing – review & editing. SK: Conceptualization, Validation, Writing – review & editing. J-HL: Conceptualization, Funding acquisition, Methodology, Project administration, Supervision, Writing – original draft, Writing – review & editing.
