# Supplementary material for: Synergizing breeding strategies via combining speed breeding, phenotypic selection, and marker-assisted backcrossing for the introgression of Glu-B1i in wheat
Source: Front Plant Sci. 2024 May 28;15:1402709. doi: 10.3389/fpls.2024.1402709 (PMC11165042; doi:10.3389/fpls.2024.1402709)
Supplement: Supplementary file 1 [file DataSheet_1.pdf]

Supplementary Material

Synergizing Breeding Strategies via Combining Speed Breeding, Phenotypic Selection, and Marker-assisted Backcrossing for the Introgression of *Glu-B1i* in Wheat

Jin-Kyung Cha[1], Hyeonjin Park[1], Youngho Kwon[1], So-Myeong Lee[1], Seong-Gyu Jang[1], Soon-Wook Kwon[2], and Jong-Hee Lee[1]*

*** Correspondence:** Jong-Hee Lee : ccriljh@korea.kr

1 Supplementary Figures and Tables

1.1 Supplementary Figures

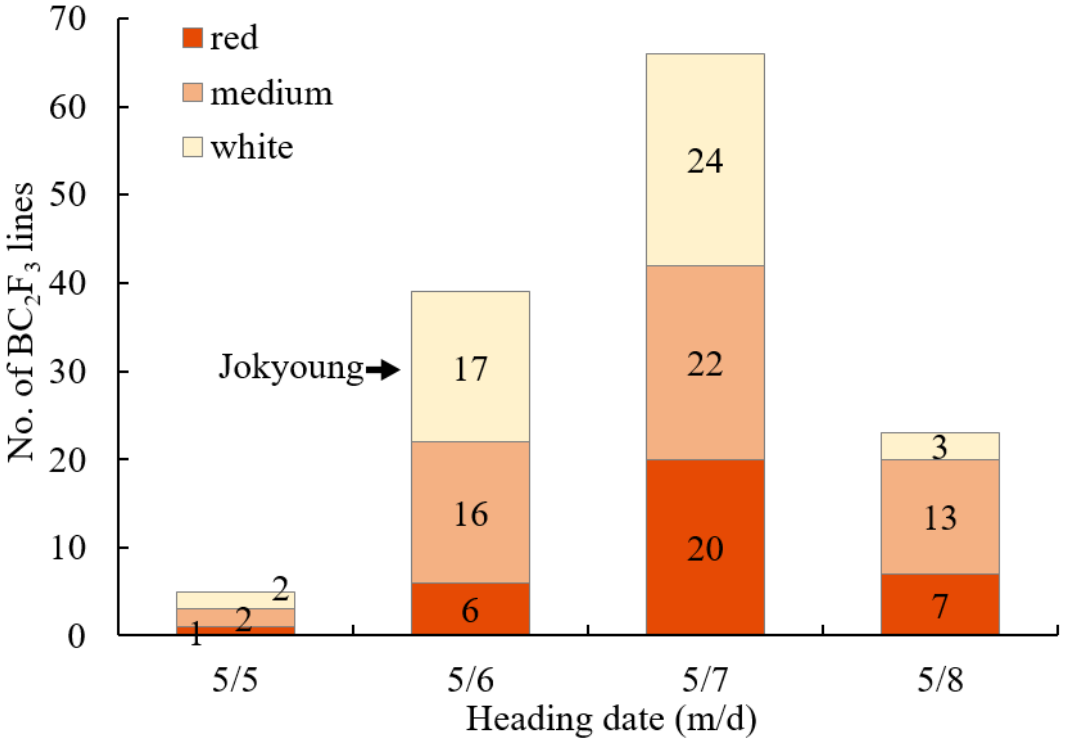

Figure S1. Distribution of Jokyoung*3/Garnet BC_2F_3 lines with different heading dates and grain colors in 2021 field conditions (spring season cultivation).

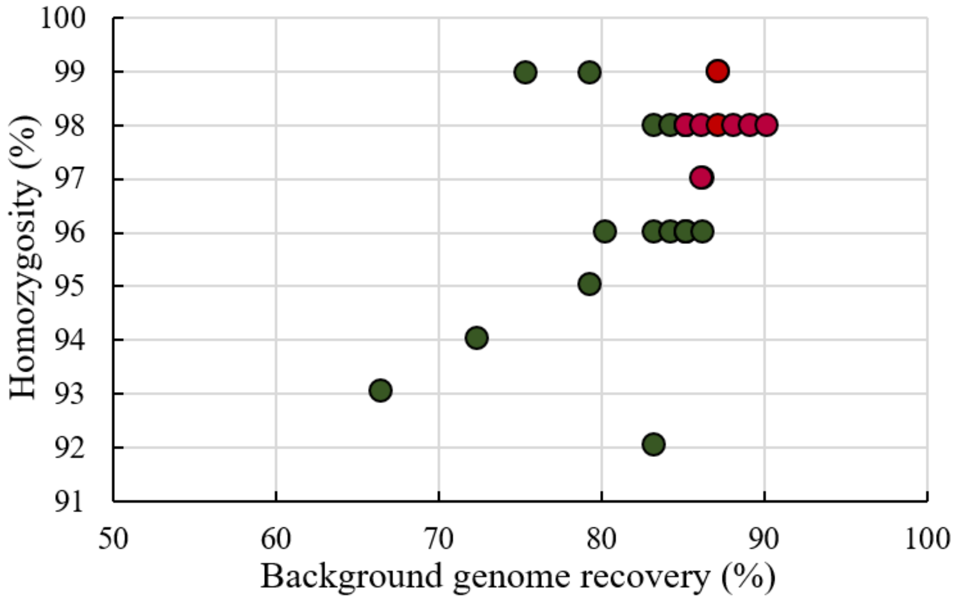

Figure S2. Distribution of background genome recovery and genome homozygosity in 27 Jokyoung*3/Garnet B_C2F_4 lines analyzed with 101 KASP assays. The red circles indicate individuals selected for the next round of background selection, which involves an additional 40 assays.

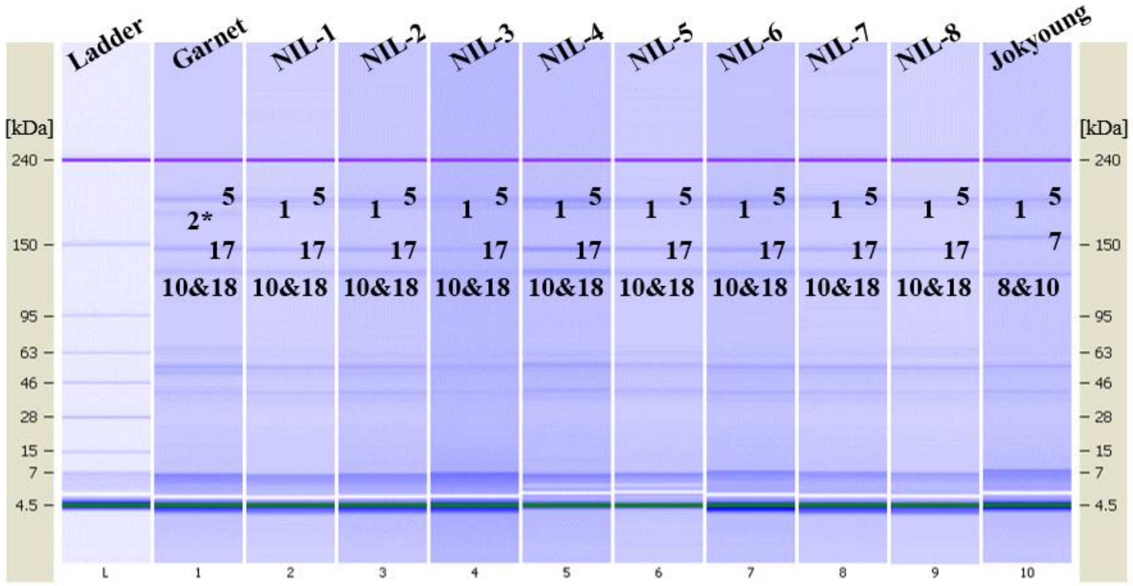

Figure S3. HMW-GS composition of eight observed yield trial lines (BC_2F_5) analyzed with lab-on-a-chip using a protein 230 assay kit (Agilent Technologies, USA).

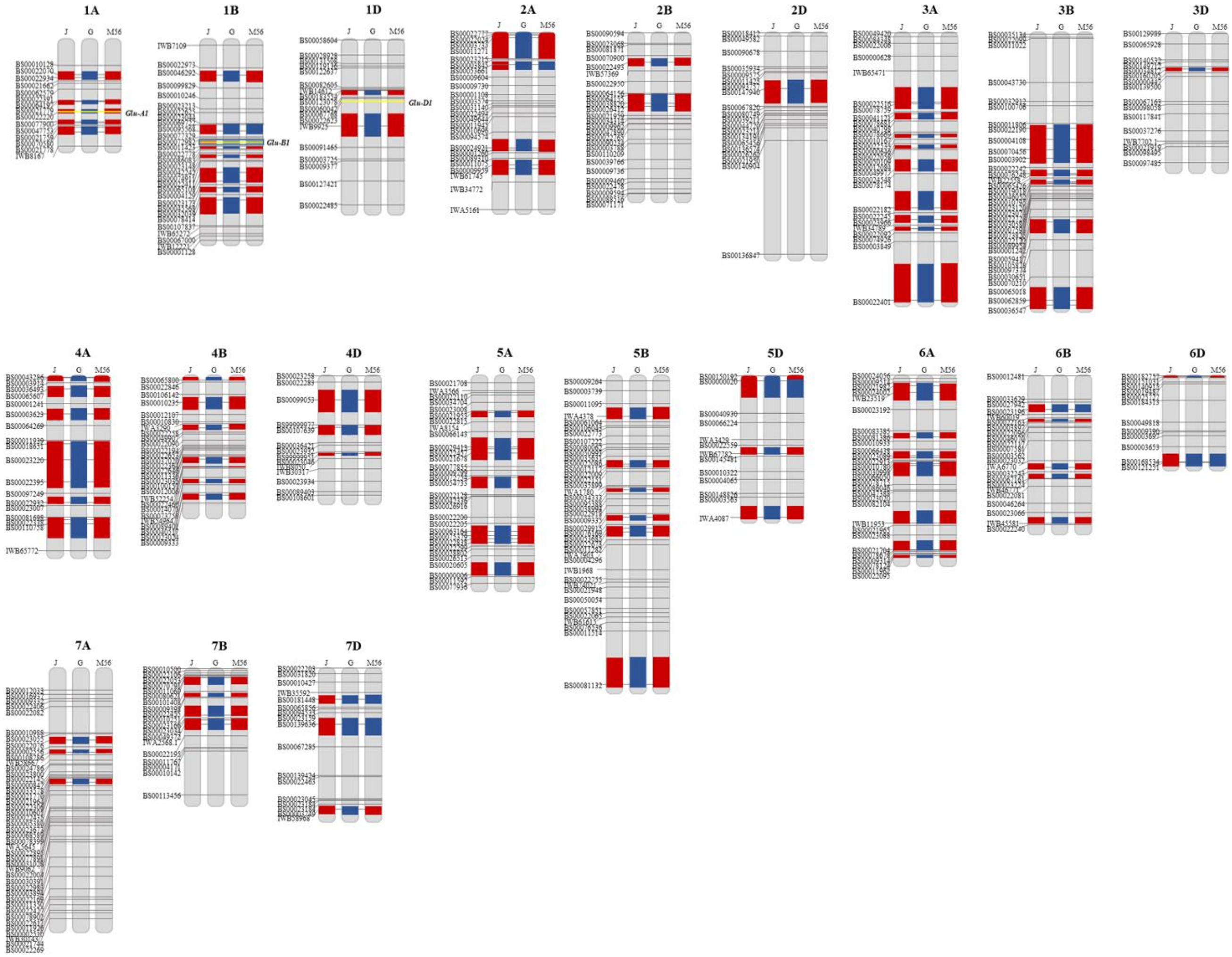

Figure S4. Graphical genotype of Milyang56 at BC_2F_4 generation and its parents evaluated with KASP assays. J: Jokyoung; G: Garnet; M56: Milyang56. The colored area indicates the polymorphic region between Jokyoung and Garnet.

1.2 Supplementary Tables

Table S1. Phenotypic and genotypic selection results in Jokyoung*3/Garnet BC_2F_2 generation.

Family no.	Family selection			Individual selection (no. of lines)		
	average DTH	DTH selection	*Glu-B1* heterozygosity	homozygous *Glu-B1i*	heterozygous	homozygous *Glu-B1b*
Jokyoung	45	target DTH	-	-	-	√
Garnet	34	-	target *Glu-B1i*	√	-	-
1	54					
2	30					
3	55					
4	28		√			
5	39					
6	28					
7	55					
8	39	√	√	21	46	14
9	65					
10	36					
11	37	√	√	30	50	27
12	41	√	√	15	19	16
13	35					
14	37					
15	39	√	√	6	12	11
16	28		√			
17	28		√			
18	34					
19	40					
20	41					
21	36	√	√	25	33	20
22	36	√	√	16	25	10
23	44					
24	41	√	√	22	45	19
25	46					
26	44					
27	36					
28	40					
29	40	√	√	34	55	35
30	67	√	√	0	2	4
31	40	√	√	26	37	17

Table S2. Summary of KASP assays used for background selection in Jokyoung*3/Garnet BC_2F_4 lines.

**Chromosome**	**No. of assays**		
	**Total tested**	**Amplified**	**Polymorphic**
1A	32	19	9
1B	40	32	11
1D	34	19	2
2A	38	30	10
2B	40	29	3
2D	30	23	2
3A	41	29	11
3B	49	36	12
3D	20	16	1
4A	32	18	10
4B	35	31	9
4D	20	15	4
5A	42	35	12
5B	55	44	8
5D	22	15	4
6A	39	29	11
6B	34	24	7
6D	16	13	3
7A	58	45	3
7B	29	20	5
7D	25	20	4
**Total**	**731**	**542**	**141**

Table S3. Observed yield trial result of eight Jokyoung-NIL-candidate lines in 2022. Different letters indicate significant differences from each other (*P*<0.001) as a result of Duncan's multiple test. HD: heading date; MD: maturity date; CL: culm length; SPL: spike length; TN: tiller number; GN: grain number; TGW: thousand-grain weight.

Line	HD (mm.dd)	MD (mm.dd)	CL (cm)		SPL (cm)		TN (no./plant)		GN (no./spike)		TGW (g)		Note
Jokyoung	04.15	05.29	65.7	b	10.7	de	17.8	c	51.4	b	57.6	a	Recurrent
Garnet	04.24	06.06	71.7	a	11.4	abc	18.6	bc	60.3	a	51.4	cd	Donor
NIL-1	04.15	05.30	67.1	b	10.7	de	19.0	bc	53.3	b	55.0	b	Milyang56
NIL-2	04.15	05.30	60.8	cd	11.7	ab	22.9	a	47.7	c	58.1	a	-
NIL-3	04.15	05.30	62.1	c	11.8	a	22.4	a	50.6	b	51.7	c	-
NIL-4	04.15	05.30	61.6	c	10.6	e	20.3	b	47.2	c	50.0	d	-
NIL-5	04.14	05.31	59.1	d	11.2	bcd	19.3	bc	46.2	c	58.4	a	-
NIL-6	04.15	05.29	67.8	b	11.8	a	18.6	bc	52.8	b	52.6	c	-
NIL-7	04.17	05.30	70.0	a	11.6	ab	17.2	c	52.9	b	55.5	b	-
NIL-8	04.14	05.29	66.4	b	11.0	cde	19.0	bc	47.9	c	58.3	a	-

Table S4. Summary of two-away ANOVA tests for observed yield trials of Jokyoung and Milyang56 in 2022 and 2023. Df: degree of freedom; SS: sum of squares; MS: mean squares.

Trait	Source	Df	SS	MS	F-value	P-value	
Culm length	Year	1	580.0	580.0	17.8	0.000	***
	Pedigree	1	0.2	0.2	0.0	0.942	
	Year×Pedigree	1	10.9	10.9	0.3	0.565	
Spike length	Year	1	5.4	5.4	10.0	0.002	**
	Pedigree	1	4.6	4.6	8.5	0.005	**
	Year×Pedigree	1	1.7	1.7	3.1	0.083	
Tiller number per plant	Year	1	45.6	45.6	3.9	0.053	
	Pedigree	1	64.1	64.1	5.5	0.023	*
	Year×Pedigree	1	80.0	80.0	6.8	0.011	*
Grain number per spike	Year	1	3020.0	3020.0	125.6	0.000	***
	Pedigree	1	93.8	93.8	3.9	0.053	
	Year×Pedigree	1	2.7	2.7	0.1	0.739	
Thousand grain weight	Year	1	164.7	164.7	287.4	0.000	***
	Pedigree	1	0.0	0.0	0.0	0.933	
	Year×Pedigree	1	19.0	19.0	33.2	0.000	***
Protein content	Year	1	3.5	3.5	2710.0	0.000	***
	Pedigree	1	2.8	2.8	2180.0	0.000	***
	Year×Pedigree	1	3.7	3.7	2902.0	0.000	***
Dry gluten content	Year	1	12.7	12.7	31.7	0.005	**
	Pedigree	1	1.6	1.6	4.1	0.114	
	Year×Pedigree	1	2.5	2.5	6.3	0.066	
Gluten index	Year	1	16.7	16.7	6.3	0.067	
	Pedigree	1	5.3	5.3	2.0	0.231	
	Year×Pedigree	1	25.3	25.3	9.5	0.037	*
SDS-sedimentation value	Year	1	32.0	32.0	128.0	0.000	***
	Pedigree	1	4.5	4.5	18.0	0.013	*
	Year×Pedigree	1	60.5	60.5	242.0	0.000	***

Table S5. Comparison of average performance of Jokyoung and Milyang56, tested as observed yield trials in 2022 and 2023. DTH: days to heading; DTM: days to maturity; CL: culm length; SPL: spike length; TN: tiller number; GN: grain number; TGW: thousand-grain weight; PC: protein content; DGC: dry gluten content; GI: gluten index; SDSS: SDS-sedimentation value.

**Name**	**DTH**	**DTM**	**CL (cm)**	**SPL (cm)**	**TN (no./plant)**	**GN (no./spike)**	**TGW (g)**	**PC (%)**	**DGC (%)**	**GI**	**SDSS (mL)**
Jokyoung	165	46	71.0	10.5	18.2	41.1	52.6	12.9	11.7	90.4	49.8
Milyang56	167	46	71.1	10.0 **	16.1 *	43.6	52.6	14.1 ***	12.6	92.0	51.3 *

Table S6. Summary of two-away ANOVA tests for trials (observed and advanced yield trial) and pedigree (Jokyoung and Milyang56) in 2023. Df: degree of freedom; SS: sum of squares; MS: mean squares.

Trait	Source	Df	SS	MS	F-value	P-value	
Days to heading	Trial	1	10.7	10.7	12.8	0.023	*
	Pedigree	1	4.5	4.5	5.4	0.081	
	Trial×Pedigree	1	13.5	13.5	16.2	0.016	*
Days to maturity	Trial	1	2.7	2.7	0.6	0.469	
	Pedigree	1	0.0	0.0	0.0	1.000	
	Trial×Pedigree	1	0.7	0.7	0.2	0.710	
Culm length	Trial	1	1490.0	1490.1	39.3	0.000	***
	Pedigree	1	61.0	61.4	1.6	0.204	
	Trial×Pedigree	1	9.0	9.2	0.2	0.623	
Spike length	Trial	1	145.4	145.4	192.1	< 2e-16	***
	Pedigree	1	33.7	33.7	44.5	0.000	***
	Trial×Pedigree	1	0.2	0.2	0.3	0.587	
Grain number per spike	Trial	1	1044.0	1044.3	25.4	0.000	***
	Pedigree	1	270.0	270.4	6.6	0.011	*
	Trial×Pedigree	1	389.0	388.8	9.5	0.002	**
Thousand grain weight	Trial	1	3.1	3.0	10.7	0.201	
	Pedigree	1	2.5	2.5	10.4	0.249	
	Trial×Pedigree	1	19.5	19.5	110.2	0.003	**
Test weight	Trial	1	175.4	175.4	100.3	0.004	**
	Pedigree	1	579.3	579.3	340.1	0.000	***
	Trial×Pedigree	1	1.8	1.8	0.1	0.747	
Protein content	Trial	1	13.7	13.7	36.6	0.000	***
	Pedigree	1	5.8	5.8	15.6	0.002	**
	Trial×Pedigree	1	2.4	2.4	6.3	0.027	**
Dry gluten content	Trial	1	10.2	10.2	110.1	0.006	**
	Pedigree	1	2.1	2.1	20.3	0.156	
	Trial×Pedigree	1	2.2	2.2	20.4	0.147	
Gluten index	Trial	1	130.1	130.1	10.8	0.202	
	Pedigree	1	824.0	824.0	110.5	0.005	**
	Trial×Pedigree	1	112.0	112.0	10.6	0.234	
SDS-sedimentation value	Trial	1	130.0	130.0	90.5	0.010	**
	Pedigree	1	280.6	280.6	200.4	0.001	***
	Trial×Pedigree	1	2.5	2.5	0.2	0.676	
